# Prognostic implications of body composition changes in patients with non-metastatic pancreatic adenocarcinoma treated with mFOLFIRINOX

**DOI:** 10.3389/fonc.2026.1762299

**Published:** 2026-03-19

**Authors:** Jong Hyuk Lee, Yousun Ko, Seongwon Na, Kyung Won Kim, Hyunseok Yoon, Changhoon Yoo, Kyu-pyo Kim, Tae Won Kim, Hyehyun Jeong, Sun Young Kim

**Affiliations:** 1 Department of Oncology, Asan Medical Center, University of Ulsan College of Medicine, Seoul, Republic of Korea; 2 Biomedical Research Center, Asan Institute for Life Sciences, Asan Medical Center, University of Ulsan College of Medicine, Seoul, Republic of Korea; 3 Department of Radiology, Asan Medical Center, University of Ulsan College of Medicine, Seoul, Republic of Korea

**Keywords:** adipose tissue, body composition, computed tomography, mFOLFIRINOX, pancreatic cancer, prognosis, skeletal muscle

## Abstract

**Background:**

Computed tomography (CT) enables non-invasive, comprehensive assessment of body composition in patients with cancer. In pancreatic ductal adenocarcinoma (PDAC), where weight and body composition change during treatment are common, serial CT evaluation may provide prognostic insights.

**Methods:**

Patients with non-metastatic PDAC treated with first-line mFOLFIRINOX between January 2017 and December 2020 were retrospectively included. Body composition at the L3 vertebral level was quantified at baseline and 12-week CT scans using a previously validated AI tool (AID-U™; iAID Inc.). Skeletal muscle area, muscle attenuation, and body fat area were used to derive skeletal muscle index (SMI), normal-to-total attenuation muscle area ratio (NAMA/TAMA), visceral fat area (VFA), and subcutaneous fat index (SFI), representing muscle mass, muscle quality, and visceral and subcutaneous adiposity, respectively.

**Results:**

A total of 733 patients with baseline CT scans were included in the analyses, and 595 patients with paired CT scans at baseline and at 12 weeks after initiation of mFOLFIRINOX were included in longitudinal change analyses. Subsequent curative resection was performed in 269 (34.6%) patients. Overall, SMI, VFA, SFI, and body mass index (BMI) declined significantly over the first 12 weeks, particularly among patients who did not undergo curative resection and in those with progressive disease. In resected patients, baseline visceral obesity was associated with worse OS. In non-resected patients, larger 12-week decreases in SMI, SFI, and BMI were associated with poorer OS.

**Conclusion:**

Serial CT-based assessment of body composition during chemotherapy may provide valuable prognostic information in non-metastatic PDAC.

## Introduction

Body composition metrics have emerged as important tools for quantifying systemic status and nutritional reserves (1–3). Computed tomography (CT)-based assessments enable comprehensive and reproducible evaluation of body composition, and it is particularly useful in patients with cancer as they frequently undergo routine CT evaluation (1, 4, 5). CT-based body composition evaluations not only measure muscle mass areas but also provide analysis of muscle attenuation, which reflects intramuscular fat deposition (myosteatosis), as well as the distribution of adipose tissue compartments (1, 4, 5).

Pancreatic ductal adenocarcinoma (PDAC) is among the most aggressive malignancies, with a five-year survival rate of less than 10% (6). Body composition is of particular interest because patients with PDAC frequently present with cachexia and malnutrition (7–10). A significant proportion of patients with PDAC experience muscle wasting during treatment, which is associated with worse prognosis (9–11). However, prior studies often analyzed heterogeneous populations including both localized and metastatic disease, and patients treated with varying treatment regimens (2, 12, 13). Given that disease burden at diagnosis and chemotherapy tolerance strongly influence survival in PDAC, evaluation in a homogeneous population is needed to clarify the prognostic role of body composition. Moreover, the implications of metrics beyond muscle mass, such as myosteatosis and adipose tissue distribution, remain incompletely defined (2, 13, 14).

In this study, we investigated the prevalence of body composition abnormalities, early post-chemotherapy changes, and their prognostic relevance in patients with non-metastatic PDAC treated with a homogeneous standard chemotherapy regimen, mFOLFIRINOX.

## Methods

### Study design and population

Patients with histologically confirmed PDAC without distant metastases who received first-line mFOLFIRINOX at Asan Medical Center, a tertiary referral hospital in Seoul, Republic of Korea, between January 2017 and December 2020, were retrospectively reviewed. Systemic therapy was delivered within a resectability-based management framework aligned with NCCN recommendations, in which treatment may be used with the aim of achieving surgical resection when feasible, or otherwise as palliative treatment (15). Patients were retrospectively stratified by subsequent curative resection status in this study.

The study protocol was approved by the Institutional Review Board of Asan Medical Center and was conducted in accordance with ethical standards of the Declaration of Helsinki. The IRB granted a waiver of informed consent for this retrospective study (IRB No. 2022-1692).

### CT images acquisition

Abdominal and pelvic CT scans were performed using Somatom Definition (Siemens Healthineers, Erlangen, Germany), Discovery CT750 HD (GE Healthcare, Milwaukee, WI), or LightSpeed VCT (GE Healthcare) scanners. Acquisition parameters were standardized as follows: 120 kVp; automated dose modulation (CareDose 4D, Siemens Healthineers; automA/smartmA, GE Healthcare); matrix size 512 × 512; and collimation 0.625 mm. Images were reconstructed with a 5-mm slice thickness using the filtered back-projection technique with the soft tissue reconstruction algorithm (B30f kernel; Siemens Healthineers Standard kernel, GE Healthcare). For contrast-enhancement, 100–150 mL of iopromide (Ultravist 370 or 300; Bayer Schering Pharma, Berlin, Germany) was administered intravenously using an automatic power injector.

### Body composition measurements

Body composition metrics were quantified using artificial intelligence software (AID-U™, iAID Inc., Seoul, Korea), previously validated for automated analysis. All measurements were performed on the portal venous phase series of abdominal and pelvic CT scans. The convolutional network-based software automatically selected the axial slice at the L3 vertebra level and segmented skeletal muscle, visceral fat, and subcutaneous fat using predetermined thresholds: -29 to +150 Hounsfield units (HU) for abdominal muscle and -190 to -30HU for adipose tissue (16, 17). The total abdominal muscle area (TAMA) was further divided by CT density into three subcategories: (1) intermuscular adipose tissue area (IMAT) (-190 to -30 HU), reflecting macroscopic fat between muscle groups and fibers; (2) normal attenuation muscle area (NAMA) (+30 to +150 HU), reflecting healthy skeletal muscle with minimal intramuscular fat; and (3) low attenuation muscle area (LAMA) (-29 to +29 HU), reflecting muscle with intramuscular lipid infiltration.

### Definition of body composition abnormalities

Four categories of body composition abnormalities were defined. Sarcopenia was assessed using the skeletal muscle index (SMI), calculated as height-adjusted skeletal muscle area (SMA/height^2^). Sarcopenia was defined using T-scores for SMI, which were calculated based on reference values from young, healthy Koreans aged 20–44 years from a large-scale cross-sectional study, as previously described (1, 18). Class I and Class II sarcopenia defined as SMI T-scores of < -1 and < -2, respectively, as previously described (1, 18). Myosteatosis was defined using the ratio of normal attenuation muscle area to total abdominal muscle area (NAMA/TAMA). Class I and Class II myosteatosis were defined as T-scores of < -1 and <-2, respectively (1). Visceral obesity was defined as a visceral fat area (VFA) ≥ 134.6 cm^2^ for men and ≥ 91.1 cm^2^ for women, based on prior large-scale Korean reference data (19). Subcutaneous obesity was defined using the subcutaneous fat index (SFI), calculated as height-adjusted subcutaneous fat area (SFA/height^2^), with thresholds of ≥ 50.0 cm²/m² for men and ≥ 42.0 cm²/m² for women (20). BMI-based obesity and underweight were defined according to WHO Asian criteria: obesity as a BMI ≥ 25.0 kg/m² and underweight as BMI < 18.5 kg/m² (21).

### Statistical analysis

Patient demographics, body composition parameters (SMI, NAMA/TAMA, VFA, SFI, BMI), and the prevalence of each abnormality (sarcopenia, myosteatosis, visceral/subcutaneous obesity, and BMI-defined obesity or underweight) were descriptively analyzed in all patients with baseline CT scans at the start of mFOLFIRINOX. Longitudinal changes in body composition parameters and abnormality prevalence during the first 12 weeks of mFOLFIRINOX were also descriptively evaluated in patients with paired abdominal CT scans obtained at baseline and at 12 weeks after treatment initiation (± 4 weeks). Changes in continuous measures were stratified into tertiles, with Tertile 1 representing the greatest decreases and Tertile 3 the smallest decreases.

Overall survival (OS) was defined as the time interval from initiation of mFOLFIRINOX to death from any cause. OS was estimated using the Kaplan–Meier method, and hazard ratios (HRs) with 95% confidence intervals (CIs) were estimated using the Cox proportional hazards models. To assess the prognostic impact of 12-week body composition changes, a landmark analysis was performed from the date of the 12-week CT scan to death from any cause (22).

All statistical analyses were conducted using R software version 4.4.0, with two-sided p-values of < 0.05 considered statistically significant.

## Results

### Patient characteristics

**Figure 1:**
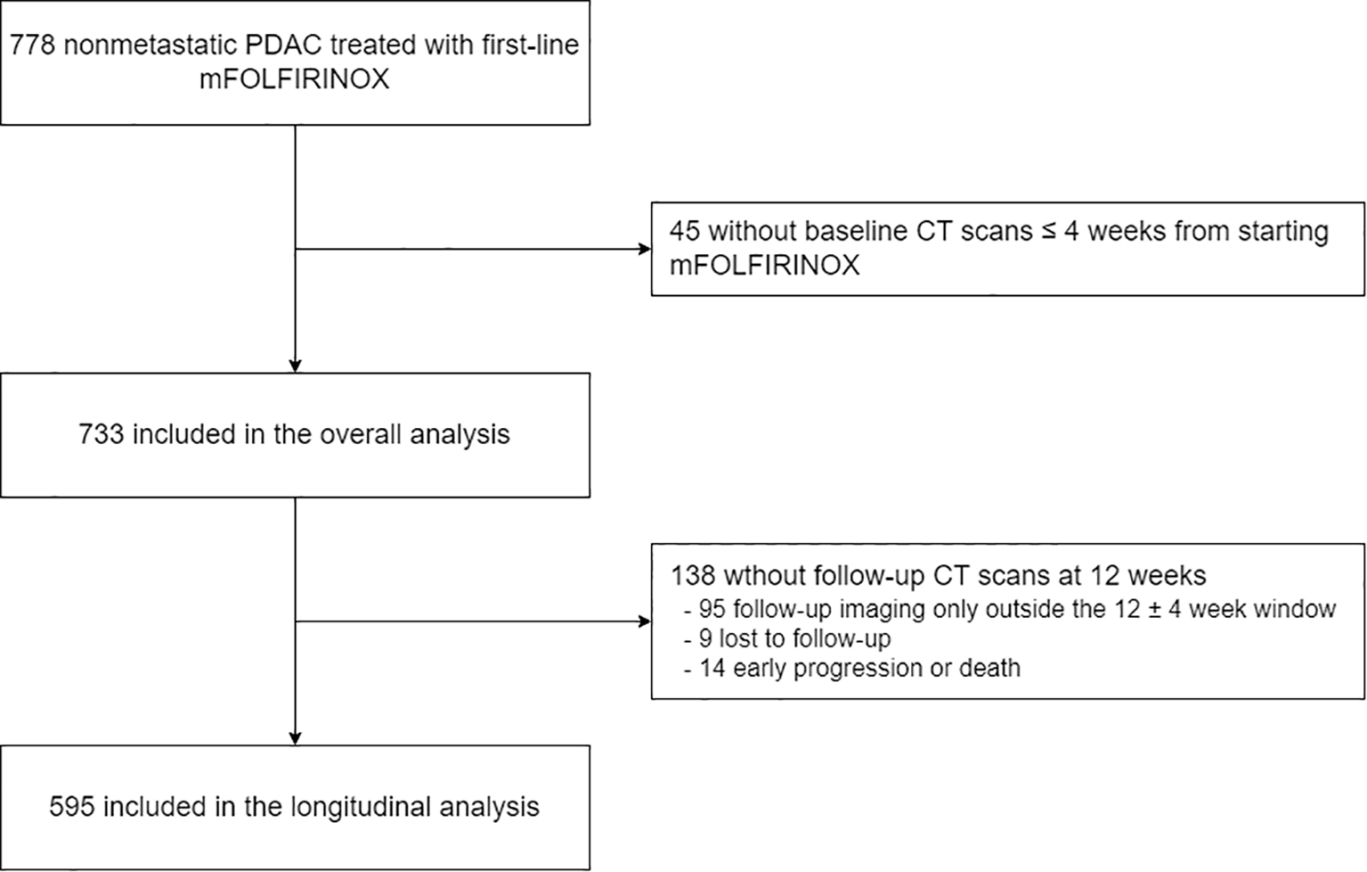
Flow diagram.

A total of 788 patients with non-metastatic PDAC who received first-line mFOLFIRINOX between January 2017 and December 2020 were retrospectively identified. Among them, 45 patients without a baseline CT scan within 4 weeks of treatment initiation were excluded, leaving 773 patients included in the analyses. Among these patients, 595 patients had paired CT scans at 12 weeks after treatment initiation (**Figure 1**).

Baseline characteristics overall patients are summarized in **Table 1**. The median age at diagnosis was 62 years (range 31–86), and 412 patients (56.2%) were male. Curative-intent surgery (R0 or R1 resection) was performed in 252 patients (34.4%). Among patients who achieved curative resection, 164 (65.1%) received adjuvant chemotherapy postoperatively. Compared with those who underwent curative-intent surgery, patients who did not had a higher proportion of locally advanced disease (55.9% vs. 14.7%) and higher CA 19–9 levels (median 141.9 vs. 95.1 U/mL) at diagnosis. At a median follow-up of 42.9 months (95% CI, 41.4–44.7), the median OS for the entire cohort was 21.6 months (95% CI, 20.2–23.5). Baseline characteristics of the 595 patients with paired CT scans are provided in **Supplementary Table 1**.

**Table 1:** Baseline characteristics of all patients.

Characteristic	All patients (N = 733)	Curative resection (n = 252)	No curative resection (n = 481)	P-value
Age (years), median (range)	62 (31–86)	62 (33–86)	63 (31–83)	0.051
Sex				0.516
Male	412 (56.2)	137 (54.4)	275 (57.2)	
Female	321 (43.8)	115 (45.6)	206 (42.8)	
ECOG performance status				0.245
0	150 (21.4)	61 (24.9)	89 (19.6)	
1	505 (72.1)	170 (69.4)	335 (65.0)	
2	45 (6.4)	14 (5.7)	31 (6.8)	
Location				0.066
Head	491 (67.4)	181 (71.8)	310 (64.4)	
Body	196 (26.7)	52 (20.6)	144 (29.9)	
Tail	34 (4.6)	15 (6.0)	19 (4.0)	
Disease status at diagnosis				< 0.001
Resectable	120 (16.4)	88 (34.9)	32 (6.7)	
Borderline resectable	307 (41.9)	127 (50.4)	180 (37.4)	
Locally advanced	306 (41.7)	37 (14.7)	269 (55.9)	
Albumin (g/dL), median (IQR)	3.6 (3.3–3.9)	3.6 (3.3–3.9)	3.6 (3.3–3.9)	0.302
CA 19-9 (U/mL), median (IQR)	116.8 (33.2–473.7)	95.1 (31.6–292.5)	141.9 (33.4–656.9)	0.007

All values are n (%) if not otherwise specified.

IQR, interquartile range; ECOG, Eastern Cooperative Oncology Group.

When comparing patients with (n = 595) and without (n = 138) paired CT scans at 12 weeks, baseline characteristics and treatment oucomes were overall comparable; however, subsequent curative resection rates were higher in the paired CT group (36.6% vs. 24.6%, *p* = 0.010) (**Suplementary Table 2**).

### Body composition abnormalities at baseline

In the overall cohort, the prevalence of any grade sarcopenia and myosteatosis at baseline was 24.8% (n = 182) and 57.0% (n = 315), respectively. Visceral obesity was observed in 35.2% (n = 258) and subcutaneous obesity in 41.7% (n = 306). By BMI, 23.3% (n = 171) were classified as obese and 6.0% (n = 44) as underweight.

Baseline prevalence of body composition abnormalities did not differ significantly between patients who underwent curative resection and those who did not, except for a marginally higher prevalence of sarcopenia in the non-surgical group (9.7% vs. 28.1%) (**Supplementary Table 3**).

### Changes in the body composition during the first 12 weeks of chemotherapy

**Figure 2:**
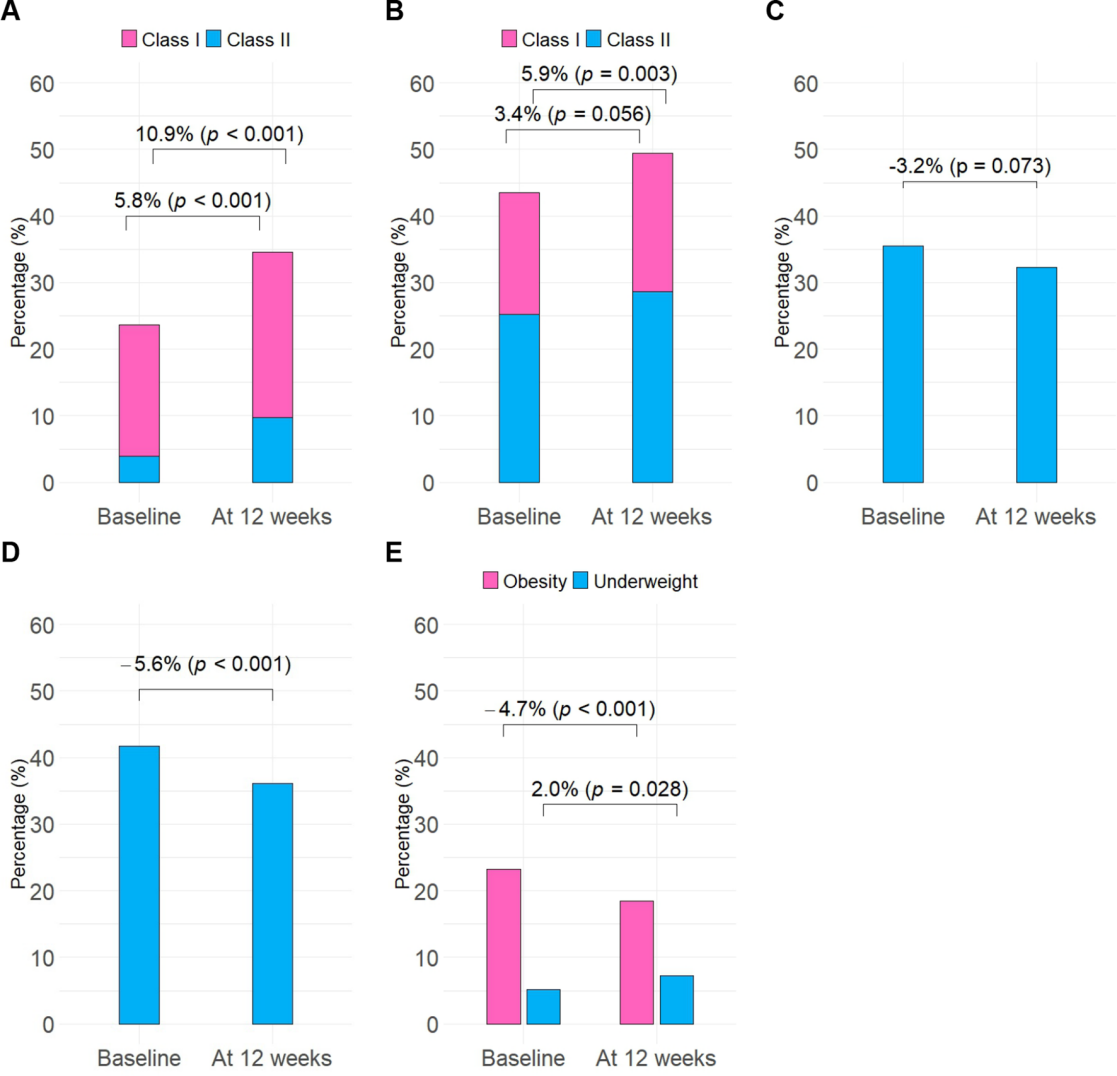
Proportions of patients with categorical body composition measures at baseline and at 12 weeks. **(A)** sarcopenia, **(B)** myosteatosis, **(C)** visceral obesity, **(D)** subcutaneous obesity, **(E)** BMI-based obesity/underweight.

During the first 12 weeks of mFOLFIRINOX, significant changes in body composition abnormalities were observed in the overall population. As shown in **Figure 2**, the prevalence of Class I and Class II sarcopenia increased by 5.1% and 5.8%, respectively, while Class I and Class II myosteatosis increased by 2.5% and 3.4%, respectively. In contrast, visceral and subcutaneous obesity declined by 4.7% and 5.6%, respectively. Similarly, BMI-defined obesity decreased by 4.7%, whereas the proportion of underweight patients increased by 2.0%. Among patients without baseline sarcopenia or myosteatosis, 87/454 (19.2%) and 83/336 (24.7%) newly developed any grade sarcopenia or myosteatosis, respectively, by week 12 (**Supplementary Figure 1**).

**Figure 3:**
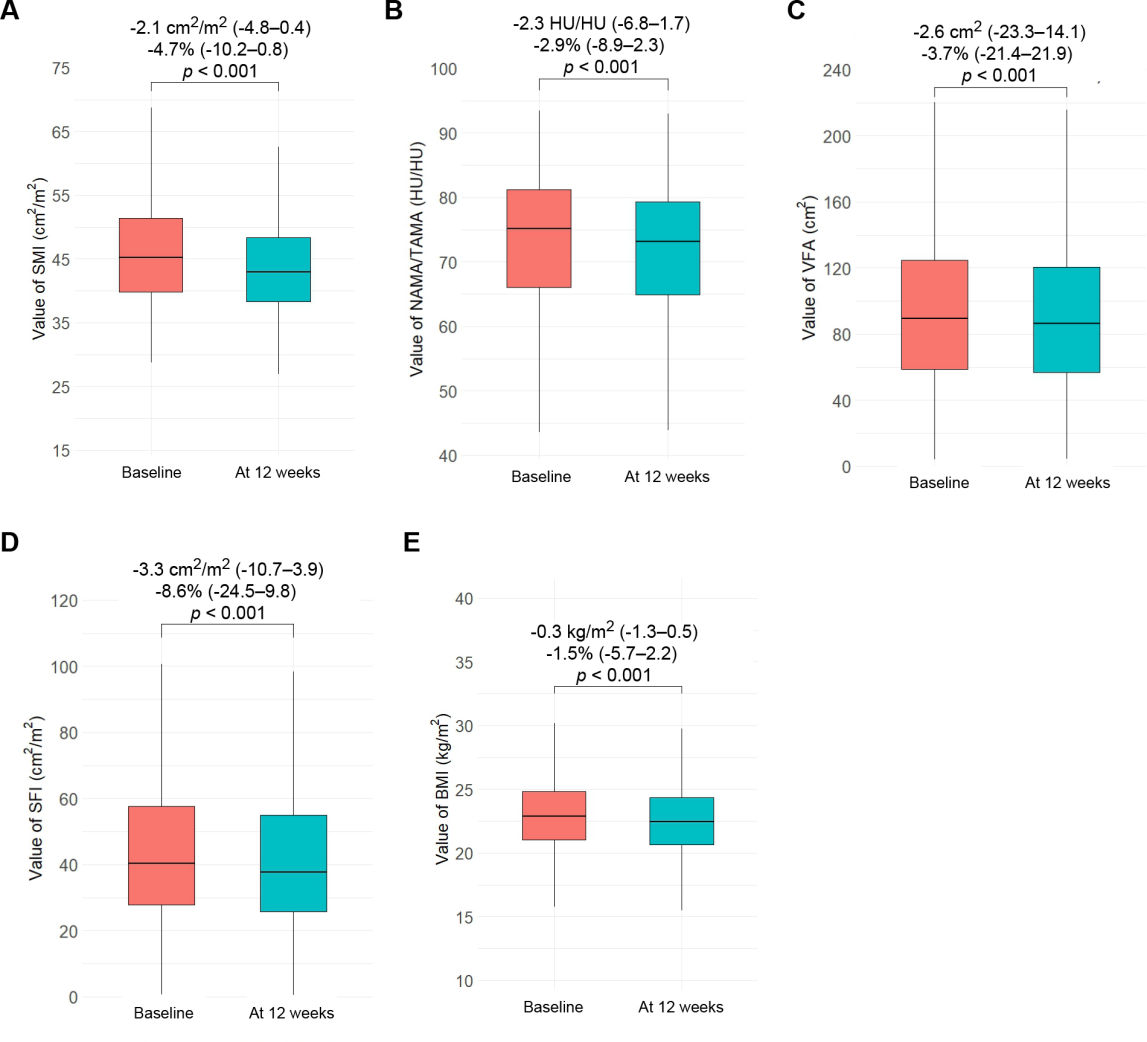
Box plots of continuous body composition values and their changes at baseline and at 12 weeks. **(A)** SMI, **(B)** NAMA/TAMA, **(C)** VFA, **(D)** SFI, **(E)** BMI. Values are presented as median (IQR). SMI, skeletal muscle index; NAMA/TAMA, normal attenuation muscle area/total abdominal muscle area; VFA, visceral fat area; SFI, subcutaneous fat index; BMI, body mass index.

Quantitative changes in continuous body composition metrics are shown in **Figure 3**. All parameters significantly declined over the 12-week period: median percentage changes were −4.7% for SMI (*p* < 0.001), −2.9% for NAMA/TAMA (*p* < 0.001), −3.7% in VFA (*p* < 0.001), −8.6% for SFI (*p* < 0.001), and −1.5% for BMI (*p* < 0.001). During the first 12 weeks, less decline in SMI, VFA, SFI, and BMI was observed in patients who subsequently underwent curative resection than in those who did not (**Supplementary Table 4**).

### Changes in the body composition by tumor response

Body composition changes during the first 12 weeks varied by treatment response. Patients with progressive disease demonstrated the greatest losses in SMI, VFA, and BMI, whereas those achieving complete or partial responses had the smallest declines (**Table 2**). In contrast, changes in the NAMA/TAMA index did not differ significantly according to tumor response (*p* = 0.292).

**Table 2:** 12-week body composition changes according to tumor response after mFOLFIRINOX.

Body composition parameter	CR + PR (N = 105)	SD (N = 447)	PD (N = 39)	P-value
SMI	-4.3% (-8.6–1.4)	-4.5% (-10.2–0.9)	-9.1% (-13.7–3.4)	0.008
NAMA/TAMA	-3.2% (-10.3–0.8)	-2.8% (-8.3–2.2)	-6.8% (-10.0–2.5)	0.292
VFA	1.6% (-15.5–34.3)	-3.2% (-21.3–17.1)	-19.2% (-34.2–4.4)	0.003
SFI	-7.8% (-21.1–13.8)	-8.2% (-24.5–10.6)	-14.2% (-33.8–3.2)	0.048
BMI	-0.7% (-5.0–2.4)	-1.4% (-5.7–2.5)	-3.8% (-9.4–0.7)	0.005

Five patients were excluded due to unknown tumor response (n = 4).

CR, complete response; PR, partial response; SD, stable disease; PD, progressive disease.

### Associations of baseline and 12-week changes in body composition with overall survival in the curative resection group

**Figure 4:**
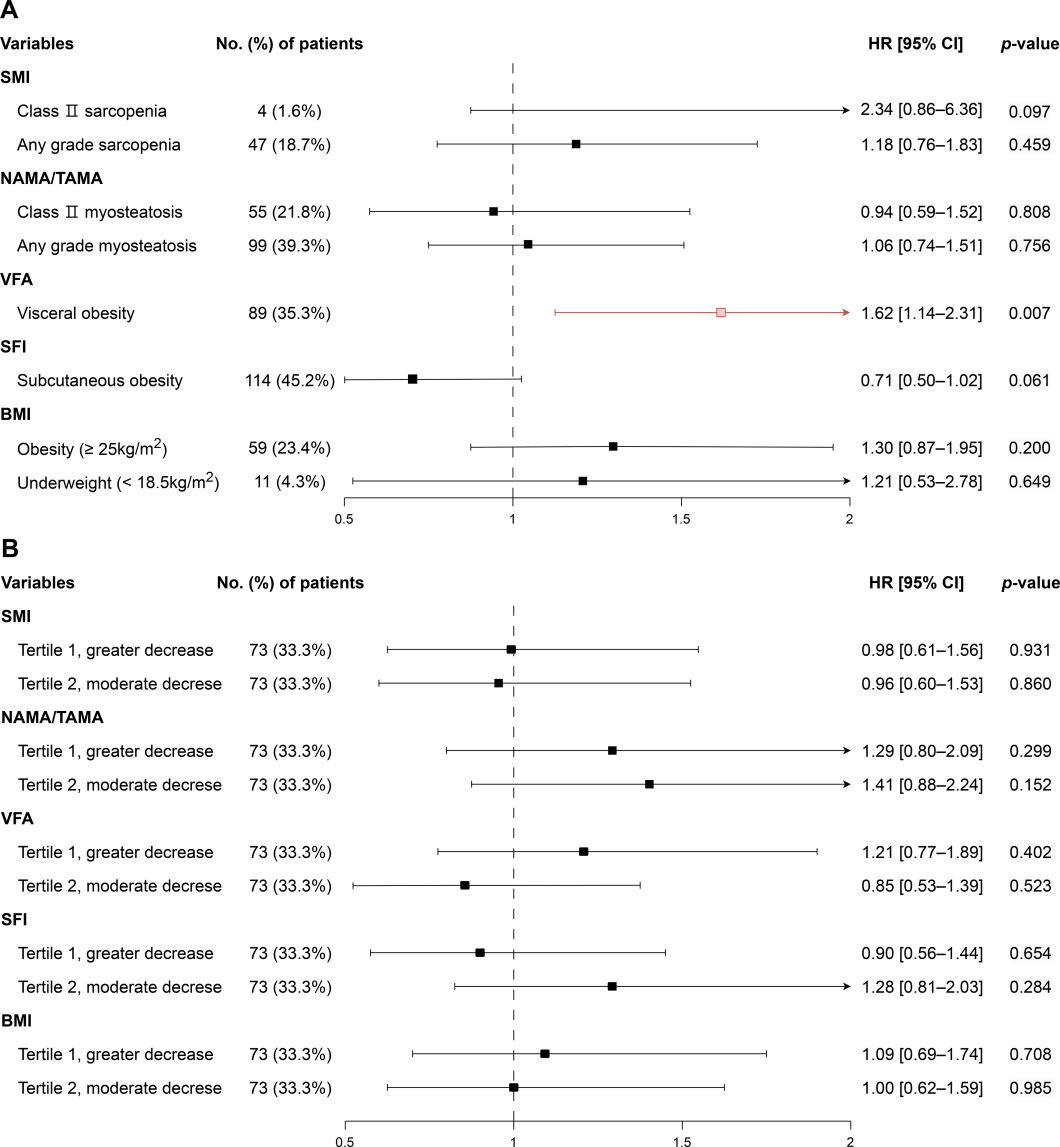
Hazard ratios for overall survival according to **(A)** baseline and **(B)** 12-week body composition changes in the curative resection group. In **(B)**, tertiles 1–3 represent changes in body composition from greater decrease to lesser decrease, with tertile 3 used as the reference. SMI, skeletal muscle index; NAMA/TAMA, normal attenuation muscle area/total abdominal muscle area; VFA, visceral fat area; SFI, subcutaneous fat index; BMI, body mass index.

Among patients who underwent curative resection, the median OS was 41.6 months (95% CI, 37.2–50.5). In this subgroup, baseline body fat distribution showed divergent prognostic implications. Visceral obesity was associated with poorer OS (HR 1.62, 95% CI, 1.14–2.31; *p* = 0.007), whereas subcutaneous obesity was marginally associated with improved OS (HR 0.71, 95% CI, 0.50–1.02; *p* = 0.061) (**Figure 4A**). Furthermore, visceral obesity was significantly associated with poorer OS in the multivariable model after adjusting for clinical variables including BMI (**Supplementary Figure 2**). Other baseline body composition abnormalities were not significantly associated with OS. With respect to early longitudinal changes, 12-week percent changes in body composition categorized by tertiles were not significantly associated with OS in the curative resection group (**Figure 4B**; **Supplementary Figure 4A**).

### Associations of baseline and 12-week changes in body composition with overall survival in the no curative resection group

**Figure 5:**
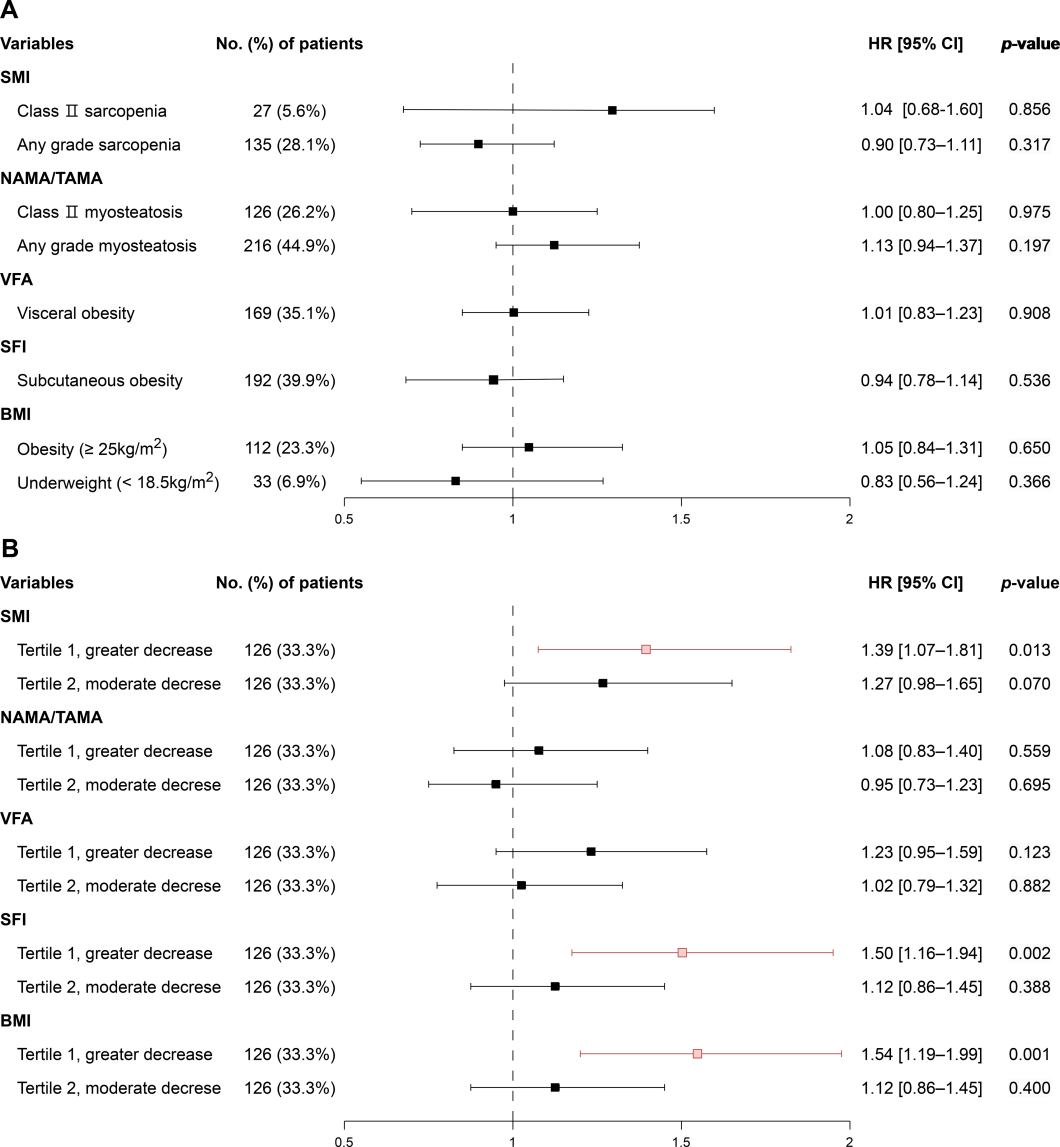
Hazard ratios for overall survival according **(A)** baseline and **(B)** 12-week body composition changes in the no curative resection group. In **(B)**, tertiles 1–3 represent changes in body composition from greater decrease to lesser decrease, with tertile 3 used as the reference. SMI, skeletal muscle index; NAMA/TAMA, normal attenuation muscle area/total abdominal muscle area; VFA, visceral fat area; SFI, subcutaneous fat index; BMI, body mass index.

In patients who did not undergo curative resection, the median OS was 17.0 months (95% CI, 15.5–18.0). Baseline body composition abnormalities were not significantly associated with OS (**Figure 5A**). By contrast, early longitudinal changes were prognostically informative. Compared with patients in Tertile 3 (minimal decline), those in Tertile 1 (greatest decline) had poorer OS for decreases in SMI (HR 1.39, 95% CI, 1.07–1.81; *p* = 0.013), SFI (HR 1.50, 95% CI, 1.16–1.94; *p* = 0.002), and BMI (HR 1.54, 95% CI, 1.19–1.99; *p* = 0.001) (**Figure 5B**; **Supplementary Figure 4B**). In the multivariable Cox regression model, greater decline in SFI remained significantly associated with poorer prognosis (HR 1.42, 95% CI, 1.03–1.96), whereas changes in BMI were not (**Supplemetnary Figure 3**).

The tertile cut-points (percent-change ranges) for each body composition parameter are provided in **Supplementary Table 5**.

## Discussion

In this study, we demonstrated that dynamic changes in body composition, including declines in muscle mass, muscle quality, body fat area, and BMI, occur during the first 12 weeks of mFOLFIRINOX in patients with non-metastatic PDAC. These changes were associated with tumor response to treatment, with greater losses observed in patients with progressive disease. Importantly, the prognostic relevance of body composition varied according to resection status. In resected patients, baseline abnormalities in body composition, particularly fat distribution, were prognostic: specifically, visceral obesity predicted worse outcomes, whereas subcutaneous obesity did not show such association. In contrast, in unresected patients, it was the changes in body composition, rather than baseline measures, that correlated with survival. Patients with pronounced declines in skeletal muscle mass and subcutaneous fat during the first 12 weeks demonstrated poorer survival.

The divergent prognostic implications according to resection status likely reflect the different clinical trajectories of these populations. Patients who did not undergo resection and continued palliative chemotherapy had markedly limited survival (median OS 17.0 months), consistent with prior studies in locally advanced unresectable PDAC (23–26). In this setting, early declines in skeletal muscle, body fat, and BMI may reflect systemic deterioration driven by aggressive disease biology and inadequate treatment response. Our findings are consistent with prior reports showing that early loss of muscle mass or weight during palliative chemotherapy is a strong predictor of poor survival (10, 27).

In contrast, among patients who achieved curative resection, baseline adiposity distribution showed different prognostic implications. The observed association between visceral obesity and poorer survival is consistent with prior studies indicating that a lower visceral-to-subcutaneous fat ratio tend to have better outcomes in PDAC (28–31), with similar patterns observed in other solid tumors (20, 32, 33). As our study cohort largely comprised patients with borderline resectable or locally advanced PDAC, those who proceeded to resection represented a selected group with a favorable treatment response. Within this subgroup, the differential prognostic implications of visceral and subcutaneous obesity may reflect differences in metabolic and inflammatory profiles. Visceral fat is metabolically active and pro-inflammatory, potentially promoting tumor progression, whereas subcutaneous fat primarily functions as an energy reserve that buffers catabolic stress (33, 34).

In our cohort, myosteatosis was not associated with prognosis. Although the adverse prognostic impact of myosteatosis in patients with cancer has been widely reported (35), its definitive diagnosis requires invasive muscle biopsy, limiting clinical applicability. CT-based muscle attenuation has therefore been used as a noninvasive surrogate (1, 5, 36, 37), but it can be influenced by contrast phase effects (36, 38). To address this limitation, we defined myosteatosis using the NAMA/TAMA ratio, a muscle area–based proportional index proposed in a large-scale Korean cohort and potentially less susceptible to contrast-related variability (1). The NAMA/TAMA ratio has been shown to decline steadily with age, reflecting age-related deterioration of muscle quality (1), and has also been associated with steatotic liver disease (39). Furthermore, T-score–based definitions of myosteatosis using the NAMA/TAMA ratio have demonstrated prognostic significance in subsequent studies of other solid tumors (40, 41).

In contrast with these prior studies with other solid cancers, the absence of an association in our study remains unclear. One explanation may be that, unlike more indolent conditions such as hepatic steatosis or malignancies with longer survival, pancreatic cancer is characterized by rapid clinical decline and profound weight loss, potentially obscuring the prognostic impact of myosteatosis. Given that PDAC is among the most cachexia-prone malignancies (7, 42), rapid catabolic deterioration during treatment is often manifested as weight loss or reductions in body fat (7, 43, 44) and may be more strongly associated with survival outcomes, although further investigation is required.

Our study has several limitations. First, it was a retrospective, single-center study and thus hypothesis-generating; validation in larger, multicenter cohorts is needed to confirm these findings and establish their relevance in routine practice. Second, body composition was assessed indirectly using CT rather than direct measurement. Nonetheless, CT-based assessment offers practical advantages, as it is routinely performed, non-invasive, and requires no additional testing. With the recent development of cachexia-directed therapies (45), CT-based body composition metrics may offer future utility as a screening tool to identify patients with poor metabolic phenotypes who could benefit from early interventions in routine clinical practice. Further studies are warranted.

Strengths of our study include a homogeneous patient population of non-metastatic PDAC, all treated with the same mFOLFIRINOX regimen. Moreover, the body composition metrics employed have been validated in Asian populations, reducing potential bias from applying criteria derived from Western cohorts, as racial differences in body composition are well recognized (21, 46–50). Furthermore, we examined not only baseline metrics but also early temporal changes during treatment, providing novel insight into the prognostic significance of body composition dynamics.

## Conclusion

In non-metastatic PDAC treated with mFOLFIRINOX, significant changes in body composition occur within the first 12 weeks and are associated with both tumor response and survival. Early loss of skeletal muscle and subcutaneous fat was associated with poorer outcomes in patients who did not undergo curative resection. Serial CT-based body composition assessment may therefore serve as a valuable prognostic tool.

## Data Availability

The data analyzed in this study is subject to the following licenses/restrictions: The clinical dataset contains sensitive patient information and cannot be made publicly available due to privacy and IRB restrictions. Requests to access these datasets should be directed to hhjeong@amc.seoul.kr, corresponding author.
