# Supplementary material for: Prognostic implications of body composition changes in patients with non-metastatic pancreatic adenocarcinoma treated with mFOLFIRINOX
Source: Front Oncol. 2026 Mar 19;16:1762299. doi: 10.3389/fonc.2026.1762299 (PMC13043377; doi:10.3389/fonc.2026.1762299)
Supplement: Supplementary file 1 [file DataSheet1.docx]

**Supplementary Material**

**Prognostic implications of body composition changes in patients with non-metastatic pancreatic adenocarcinoma treated with mFOLFIRINOX**

**Authors:**

Jong Hyuk Lee^1^, Yousun Ko^2^, Seongwon Na^2^, Kyung Won Kim^2,3^, Hyunseok Yoon^1^, Changhoon Yoo^1^, Kyu-pyo Kim^1^, Tae Won Kim^1^, Hyehyun Jeong^1*^, Sun Young Kim^1*^

^1^Department of Oncology, Asan Medical Center, University of Ulsan College of Medicine, Seoul, Republic of Korea

^2^Biomedical Research Center, Asan Institute for Life Sciences, Asan Medical Center, University of Ulsan College of Medicine, Seoul, Republic of Korea

^3^Department of Radiology, Asan Medical Center, University of Ulsan College of Medicine, Seoul, Republic of Korea

**Correspondence:**

Sun Young Kim, MD, PhD

Department of Oncology, Asan Medical Center, University of Ulsan College of Medicine

88, Olympic-ro 43-gil, Songpa-gu, Seoul 05505, Republic of Korea

Tel: +82-2-3010-3204

Fax: +82-2-3010-6981

E-mail: sunyoungkim@amc.seoul.kr

Hyehyun Jeong, MD, PhD

Department of Oncology, Asan Medical Center, University of Ulsan College of Medicine

88, Olympic-ro 43-gil, Songpa-gu, Seoul 05505, Republic of Korea

Tel: +82-2-3010-0999

Fax: +82-2-3010-6981

E-mail: hhjeong@amc.seoul.kr

**Supplementary Table 1. Baseline characteristics of patients with paired CT scans**

|  | **All patients**  **(N = 595)** | **Curative resection**  **(n = 218)** | **No curative resection**  **(n = 377)** | **p-value** |
| --- | --- | --- | --- | --- |
| **Age (years), median (range)** | 62 (31–86) | 61 (33–86) | 63 (31–81) | 0.072 |
| **Sex** |  |  |  | 0.591 |
| Male | 332 (55.8) | 118 (54.1) | 214 (56.8) |  |
| Female | 263 (44.2) | 100 (45.9) | 163 (43.2) |  |
| **ECOG performance status** |  |  |  | 0.248 |
| 0 | 111 (19.6) | 49 (23.1) | 62 (17.5) |  |
| 1 | 420 (74.1) | 151 (71.2) | 269 (75.8) |  |
| 2 | 36 (6.3) | 12 (5.7) | 24 (6.8) |  |
| **Location** |  |  |  | 0.255 |
| Head | 412 (69.2) | 158 (71.2) | 254 (67.4) |  |
| Body | 148 (24.9) | 45 (20.6) | 103 (27.3) |  |
| Tail | 28 (4.7) | 13 (6.0) | 15 (4.0) |  |
| **Disease status at diagnosis** |  |  |  | < 0.001 |
| Resectable | 95 (16.0) | 71 (32.6) | 24 (6.4) |  |
| Borderline resectable | 259 (43.5) | 112 (51.4) | 147 (39.0) |  |
| Locally advanced | 241 (40.5) | 35 (16.1) | 206 (54.6) |  |
| **Albumin (g/dL), median (IQR)** | 3.6 (3.3–3.9) | 3.6 (3.3–3.9) | 3.6 (3.3–3.9) | 0.388 |
| **CA 19-9 (U/mL),**  **median (IQR)** | 123.9  (34.3–489.9) | 92.5  (32.6–278.0) | 170.5  (37.0–717.8) | 0.001 |

Note: All values are n (%) if not otherwise specified.

Abbreviations: IQR, interquartile range; ECOG, Eastern Cooperative Oncology Group.

**Supplementary Table 2. Baseline characteristics and treatment outcomes of patients with and without paired CT scans**

|  | **All patients**  **(N = 733)** | **Patients with paired CT scans**  **(N = 595)** | **Patients without paired CT scans**  **(n = 138)** | **p-value** |
| --- | --- | --- | --- | --- |
| **Age (years), median (range)** | 62 (31–86) | 62 (31–86) | 63 (40–83) | 0.242 |
| **Sex** |  |  |  | 0.713 |
| Male | 412 (56.2) | 332 (55.8) | 80 (58.0) |  |
| Female | 321 (43.8) | 263 (44.2) | 58 (42.0) |  |
| **ECOG performance status** |  |  |  | 0.042 |
| 0 | 150 (21.4) | 111 (19.6) | 39 (29.3) |  |
| 1 | 505 (72.1) | 420 (74.1) | 85 (63.9) |  |
| 2 | 45 (6.4) | 36 (6.3) | 9 (6.8) |  |
| **Location** |  |  |  | 0.014 |
| Head | 491 (67.4) | 412 (69.2) | 79 (57.2) |  |
| Body | 196 (26.7) | 148 (24.9) | 48 (34.8) |  |
| Tail | 34 (4.6) | 28 (4.7) | 6 (4.3) |  |
| **Disease status at diagnosis** |  |  |  | 0.171 |
| Resectable | 120 (16.4) | 95 (16.0) | 25 (18.1) |  |
| Borderline resectable | 307 (41.9) | 259 (43.5) | 48 (34.8) |  |
| Locally advanced | 306 (41.7) | 241 (40.5) | 65 (47.1) |  |
| **Albumin (g/dL), median (IQR)** | 3.6 (3.3–3.9) | 3.6 (3.3–3.9) | 3.6 (3.3–3.9) | 0.391 |
| **CA 19-9 (U/mL),**  **median (IQR)** | 116.8  (33.2–473.7) | 123.9  (34.3–489.9) | 90.6  (25.0–395.5) | 0.168 |
| **Curative resection** |  |  |  | 0.010 |
| Yes | 252 (34.4) | 218 (36.6) | 34 (24.6) |  |
| No | 481 (65.6) | 377 (63.4) | 104 (75.4) |  |
| **Tumor response** |  |  |  | 0.144 |
| CR+PR | 124 (17.5) | 105 (17.8) | 19 (16.0) |  |
| SD | 533 (75.1) | 447 (75.6) | 86 (72.3) |  |
| PD | 53 (7.5) | 39 (6.6) | 14 (11.8) |  |
| **OS (months), 95% CI** | 21.6  (20.2–23.5) | 21.7  (20.2–23.8) | 20.7  (16.9–24.8) | 0.167 |

Note: All values are n (%) if not otherwise specified.

Abbreviations: IQR, interquartile range; ECOG, Eastern Cooperative Oncology Group; CR, complete response; PR, partial response; SD, stable disease; PD, progressive disease; OS, Overall Survival; CI, confidence interval.

**Supplementary Table 3. Comparison of baseline body composition measures between patients with and without curative resection**

|  | **All patients**  **(N = 733)** | **Curative resection**  **(n = 252)** | **No curative resection**  **(n = 481)** | **p-value** |
| --- | --- | --- | --- | --- |
| **SMI (cm^2^/m^2^)** | 45.15  (39.72–50.79) | 45.76  (40.29–51.98) | 45.06  (39.50–50.21) | 0.267 |
| T-score | -0.23  (-0.90–0.59) | -0.09  (-0.80–0.61) | -0.31  (-1.09–0.55) | 0.034 |
| Class I sarcopenia | 151 (20.6) | 43 (17.1) | 108 (22.5) | 0.106 |
| Class II sarcopenia | 31 (4.2) | 4 (1.6) | 27 (5.6) | 0.017 |
| Any grade sarcopenia | 182 (24.8) | 47 (9.7) | 135 (28.1) | 0.007 |
| **NAMA/TAMA (cm^2^/cm^2^)** | 74.86  (66.00–81.29) | 75.37  (67.54–81.73) | 74.70  (64.89–81.20) | 0.355 |
| T-score | -0.70  (-1.91–0.21) | -0.62  (-1.73–0.25) | -0.75  (-2.07–0.18) | 0.320 |
| Class I myosteatosis | 134 (18.3) | 44 (17.5) | 90 (18.7) | 0.752 |
| Class II myosteatosis | 181 (24.7) | 55 (21.8) | 126 (26.2) | 0.225 |
| Any grade myosteatosis | 315 (57.0) | 99 (39.3) | 265 (55.1) | 0.167 |
| **VFA (cm^2^)** | 91.64  (58.60–128.05) | 94.43  (61.34–130.86) | 90.04  (57.67–127.24) | 0.461 |
| Visceral obesity | 258 (35.2) | 89 (35.3) | 169 (35.1) | 1.000 |
| **SFI (cm^2^/m^2^)** | 40.55  (27.89–57.62) | 43.40  (30.39–58.71) | 39.71  (27.35–56.57) | 0.114 |
| Subcutaneous obesity | 306 (41.7) | 114 (45.2) | 192 (39.9) | 0.191 |
| **BMI (kg/m^2^)** | 22.81  (20.97–24.84) | 22.91  (21.14–24.85) | 22.70  (20.71–24.84) | 0.326 |
| Obesity | 171 (23.3) | 59 (23.4) | 112 (23.3) | 1.000 |
| Underweight | 44 (6.0) | 11 (4.4) | 33 (6.9) | 0.235 |

Data are n (%) unless otherwise indicated.

Abbreviations: SMI, skeletal muscle index; NAMA/TAMA, normal attenuation muscle area/total abdominal muscle area; VFA, visceral fat area; SFI, subcutaneous fat index; BMI, body mass index.

**Supplementary Table 4. Comparison of body composition changes between baseline and 12 weeks**

|  | **All patients**  **(N = 595)** | **Curative resection**  **(n = 218)** | **No curative resection**  **(n = 377)** | **p-value** |
| --- | --- | --- | --- | --- |
| **SMI (cm^2^/m^2^)** | -4.7%  (-10.2%–0.8%) | -3.2%  (-8.4%–1.7%) | -6.0%  (-11.1%–0.4%) | 0.001 |
| T-score | -0.35  (-0.76–0.06) | -0.25  (-0.63–0.12) | -0.43  (-0.87–0.03) | 0.002 |
| Class I sarcopenia | 5.1% | 6.9% | 4.0% | 1.000 |
| Class II sarcopenia | 5.8% | 3.2% | 7.4% | 1.000 |
| Any grade sarcopenia | 10.9% | 10.1% | 11.4% | 1.000 |
| **NAMA/TAMA (cm^2^/cm^2^)** | -2.9%  (-8.9%–2.3%) | -3.2%  (-9.0–1.2%) | -2.8%  (-8.7%–3.2%) | 0.164 |
| T-score | -0.34  (-0.99–0.25) | -0.38  (-1.02–0.12) | -0.30  (-0.96–0.31) | 0.172 |
| Class I myosteatosis | 2.5% | 6.0% | 0.5% | 1.000 |
| Class II myosteatosis | 3.4% | 6.0% | 1.9% | 1.000 |
| Any grade myosteatosis | 5.9% | 11.9% | 2.4% | 1.000 |
| **VFA (cm^2^)** | -3.7%  (-21.4%–21.9%) | 0.7%  (-15.6%–25.7%) | -6.1%  (-23.7%–18.6%) | 0.030 |
| Visceral obesity | -4.7% | -4.6% | -4.8% | 1.000 |
| **SFI (cm^2^/m^2^)** | -8.6%  (-24.5%–9.8%) | -5.0%  (-19.6%–13.0%) | -13.3%  (-27.5%–7.3%) | < 0.001 |
| Subcutaneous obesity | -5.6% | -2.8% | -7.2% | 1.000 |
| **BMI (kg/m^2^)** | -1.5%  (-5.7%–2.2%) | -0.2%  (-4.1%–3.4%) | -2.2%  (-6.8%–1.5%) | < 0.001 |
| Obesity | -4.7% | -2.3% | -6.1% | 1.000 |
| Underweight | 2.0% | 1.8% | 2.1% | 1.000 |

Note: Summarizes the changes in prevalence of categorical measures (e.g., sarcopenia, myosteatosis) and quantitative continuous variables (e.g., SMI, NAMA/TAMA, T-scores), comparing curative vs. non-curative resection groups.

Abbreviations: SMI, skeletal muscle index; NAMA/TAMA, normal attenuation muscle area/total abdominal muscle area; VFA, visceral fat area; SFI, subcutaneous fat index; BMI, body mass index.

**Supplementary Table 5. Tertiles of changes in body composition**

|  |  | **All patients**  **(N = 595)** | **Curative resection**  **(n = 218)** | **No curative resection**  **(n = 377)** | **p-value** |
| --- | --- | --- | --- | --- | --- |
| **SMI** | Tertile 1  Tertile 2  Tertile 3 | -12.8%  (-16.3%–-10.2%)  -4.7%  (-6.7%–-2.7%)  3.6%  (0.9%–9.1%) | -12.1%  (-13.9%–-10.5%)  -4.7%  (-6.7%–-2.9%)  3.4%  (0.9%–9.9%) | -13.2%  (-17.6%–-10.1%)  -4.7%  (-6.6%--2.7%)  4.0%  (-6.3%–2.7%) | 0.003 |
| **NAMA/TAMA** | Tertile 1  Tertile 2  Tertile 3 | -11.8%  (-15.7%–-8.9%)  -2.8%  (-4.4%–-1.3%)  5.5%  (2.3%–10.7%) | -11.3%  (-14.9%–-8.9%)  -2.9%  (-4.6%–-1.7%)  3.8%  (1.8%–8.4%) | -11.9%  (-16.6%–-8.7%)  -2.8%  (-4.0%–-1.3%)  6.4%  (2.8%–11.9%) | 0.448 |
| **VFA** | Tertile 1  Tertile 2  Tertile 3 | -28.8%  (-39.8%–-21.4%)  -3.5%  (-10.8%–1.9%)  34.0%  (22.0%–60.4%) | -33.1%  (-40.9%–-21.6%)  -3.0%  (-10.8%–2.4%)  31.2%  (23.3%–46.2%) | -28.3%  (-39.6%–-21.3%)  -3.8%  (-10.4%–1.7%)  36.8%  (21.8%–75.0%) | 0.002 |
| **SFI** | Tertile 1  Tertile 2  Tertile 3 | -32.6%  (-42.5%–-24.5%)  -8.6%  (-14.5%–-3.3%)  23.9%  (10.0%–46.9%) | -30.6%  (-39.3%–-24.4%)  -8.0%  (-13.4%–-2.9%)  24.7%  (11.4%–50.2%) | -33.1%  (-43.8%–-24.6%)  -8.9%  (-14.9%–-3.3%)  23.8%  (8.8%–42.9%) | 0.003 |
| **BMI** | Tertile 1  Tertile 2  Tertile 3 | -7.5%  (-9.8%–-5.7%)  -1.5%  (-2.8%–-0.3%)  4.7%  (2.2%–7.5%) | -6.3%  (-9.0%–-5.3%)  -1.8%  (-2.9%–-0.3%)  4.7%  (2.2%–7.5%) | -7.9%  (-9.9%–-6.0%)  -1.4%  (-2.7%–-0.4%)  4.6%  (2.5%–7.6%) | < 0.001 |

Note: Lists the median (Q1–Q3) of body composition changes across the three tertiles (as used in Figures 4, 5, and Supplementary Figure 1) and compares the distributions (p-values) between curative and non-curative resection groups.

Abbreviations: SMI, skeletal muscle index; NAMA/TAMA, normal attenuation muscle area/total abdominal muscle area; VFA, visceral fat area; SFI, subcutaneous fat index; BMI, body mass index.

**Supplementary Figure 1. Changes in the body composition abnormality categories during the first 12 weeks, for (A) sarcopenia, (B) myosteatosis, (C) visceral obesity, (D) subcutaneous obesity, (E) BMI categories.
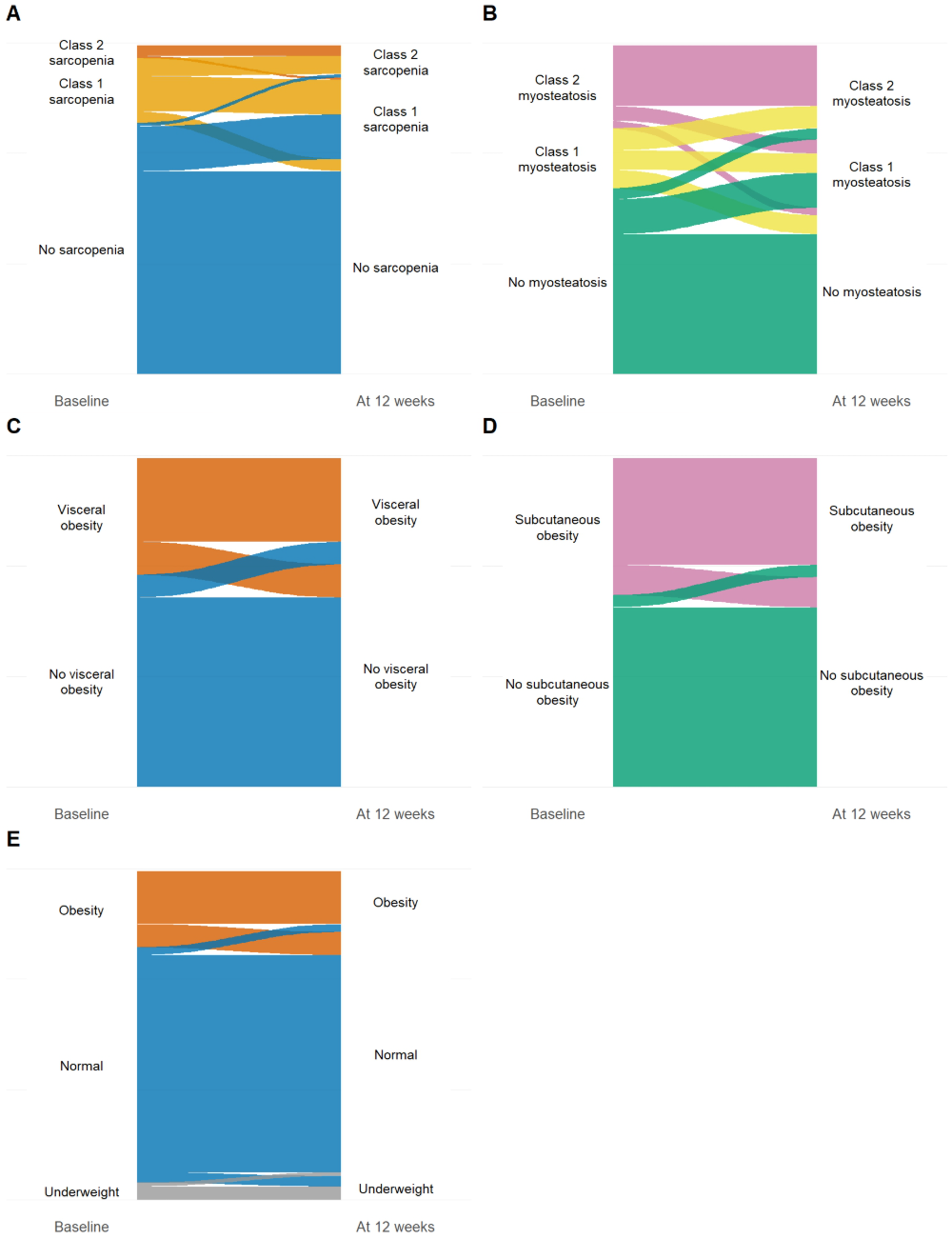
**

**Supplementary Figure 2. Multivariable Cox model for overall survival in the curative resection group, according to (A) baseline body composition, (B) 12-week body composition changes.**

**
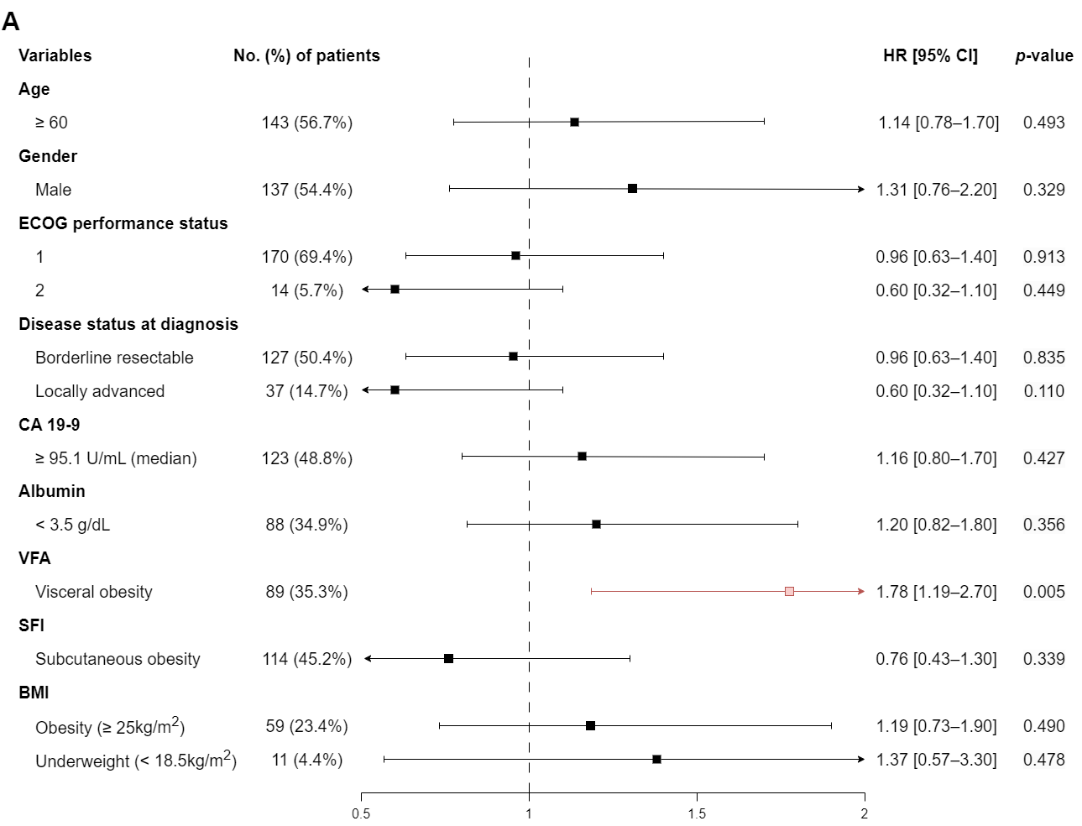

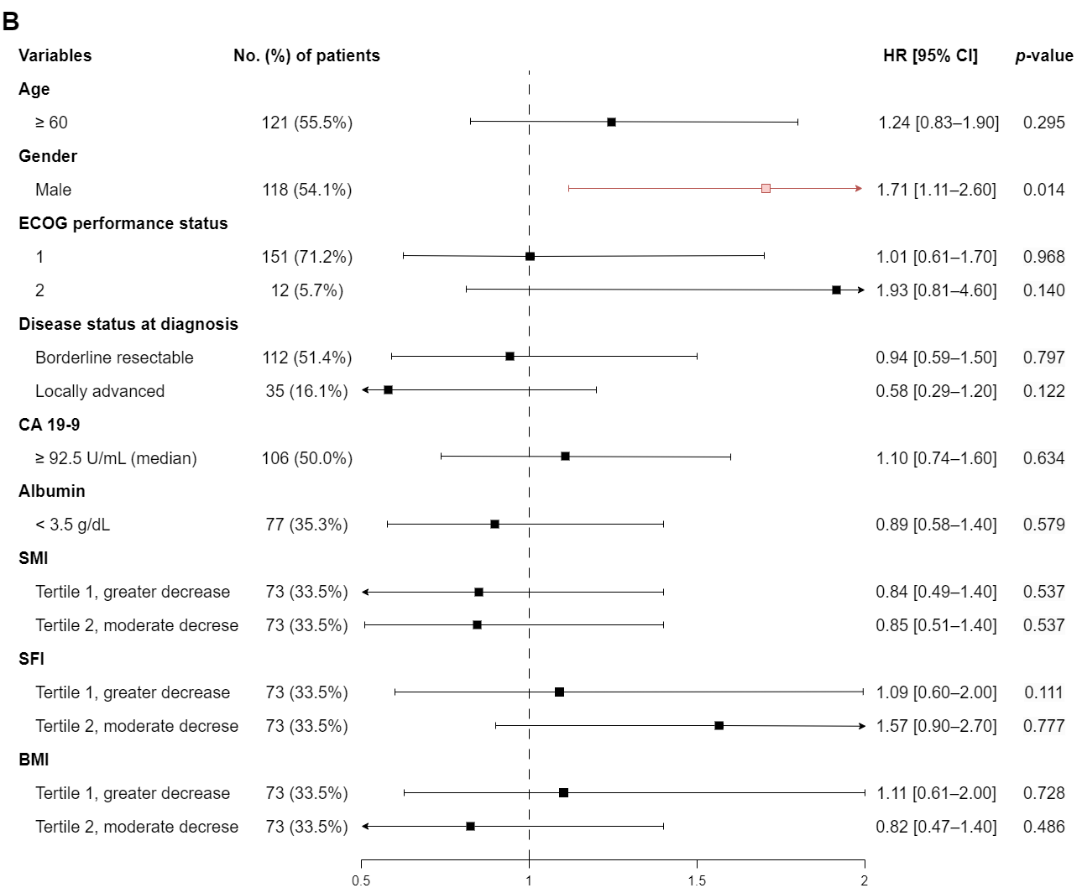
**

Note: Compares hazard ratios of Tertile 1 (greater decrease) and Tertile 2 (moderate decrease) against Tertile 3 (lesser decrease) for each body composition variable. For analyses of 12-week body composition changes, OS was calculated from the follow-up CT at 12 weeks.

Abbreviation: ECOG, Eastern Cooperative Oncology Group; SMI, skeletal muscle index; SFI, subcutaneous fat index; BMI, body mass index.

**Supplementary Figure 3. Multivariable Cox model for overall survival in the no curative resection group, according to (A) baseline body composition, (B) 12-week body composition changes.**

**
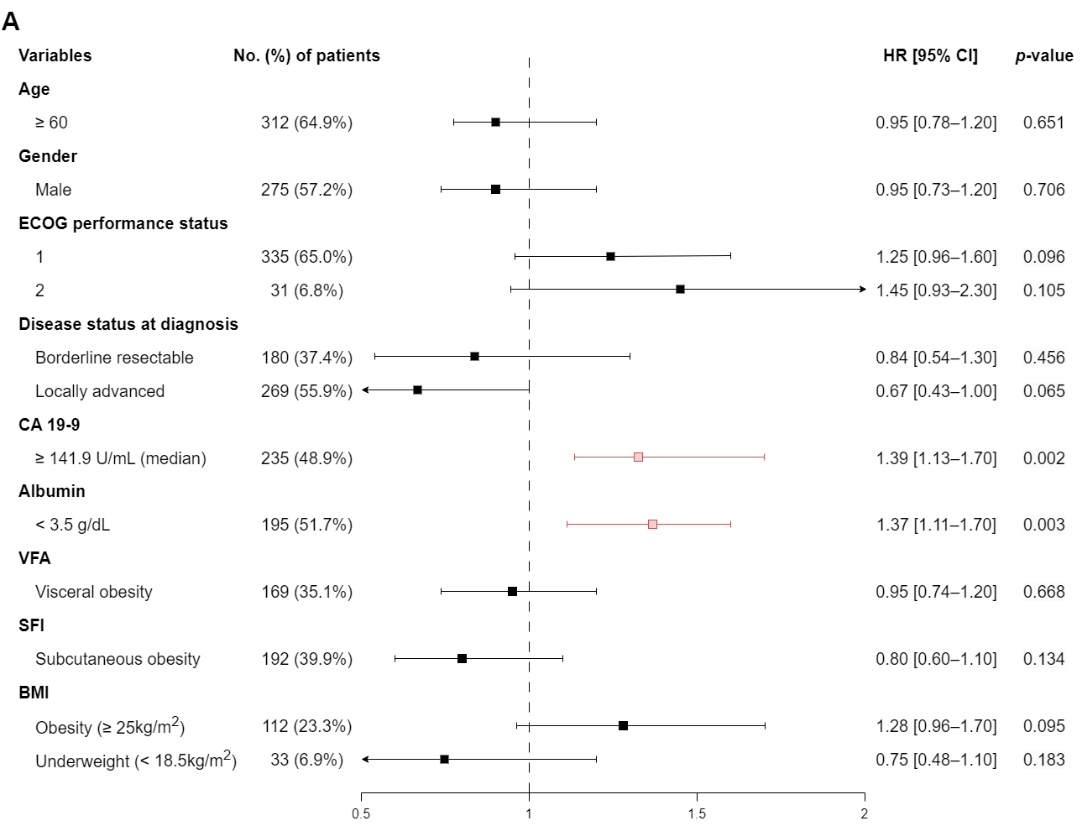

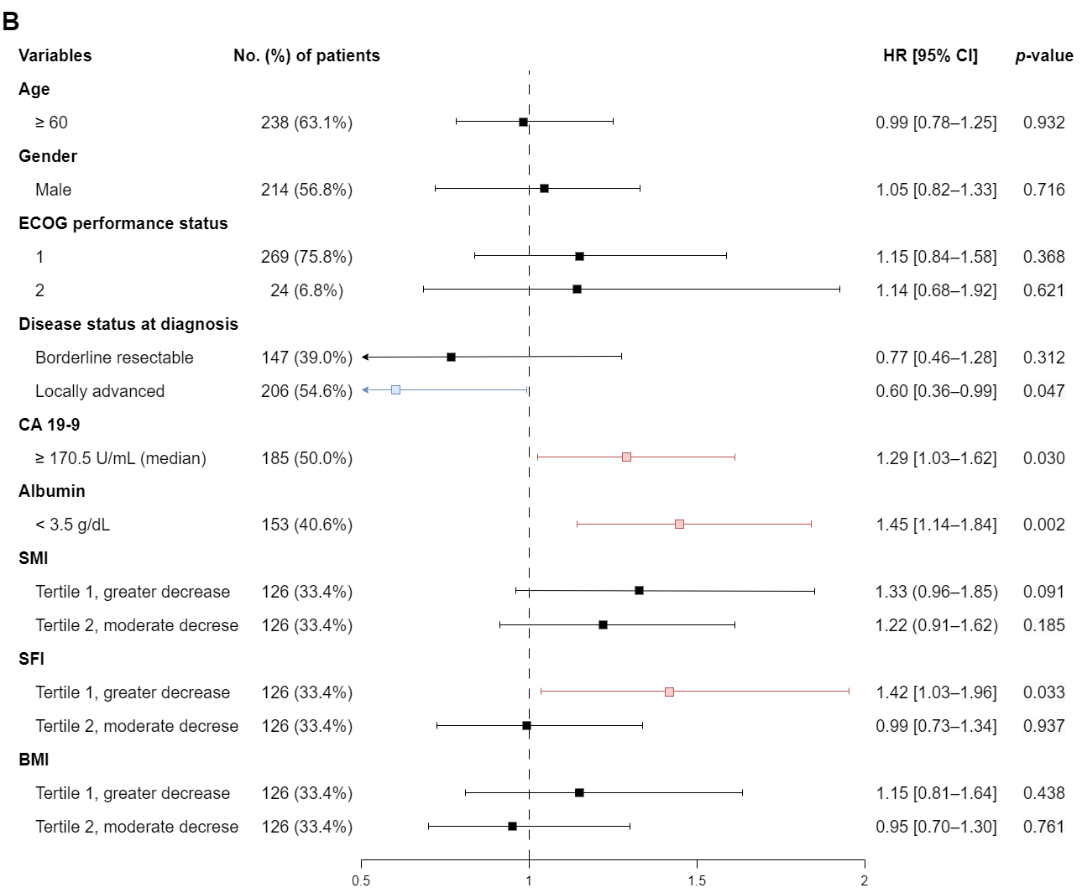
**

Note: Compares hazard ratios of Tertile 1 (greater decrease) and Tertile 2 (moderate decrease) against Tertile 3 (lesser decrease) for each body composition variable. For analyses of 12-week body composition changes, OS was calculated from the follow-up CT at 12 weeks.

Abbreviation: ECOG, Eastern Cooperative Oncology Group; SMI, skeletal muscle index; SFI, subcutaneous fat index; BMI, body mass index.


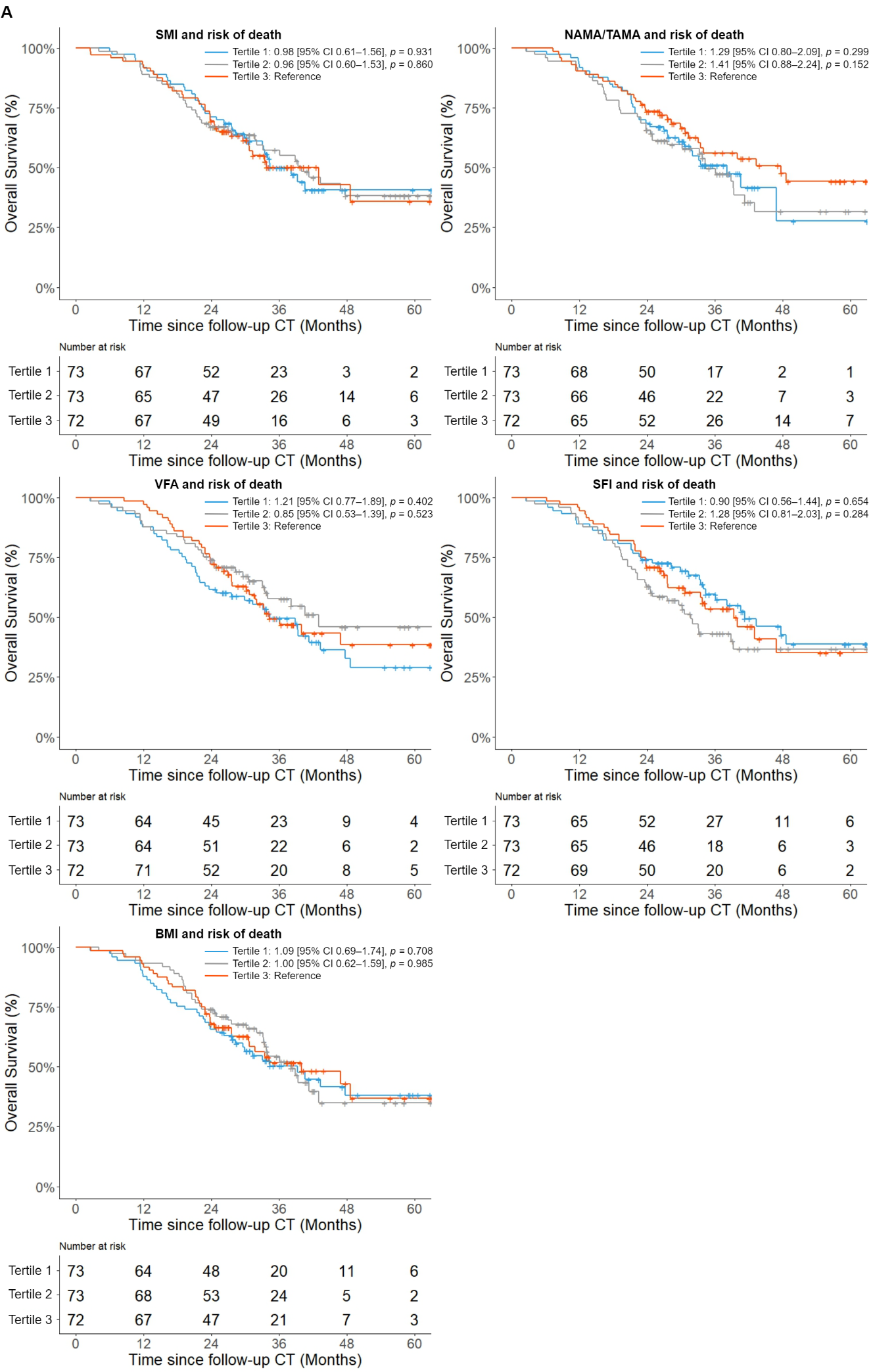


**
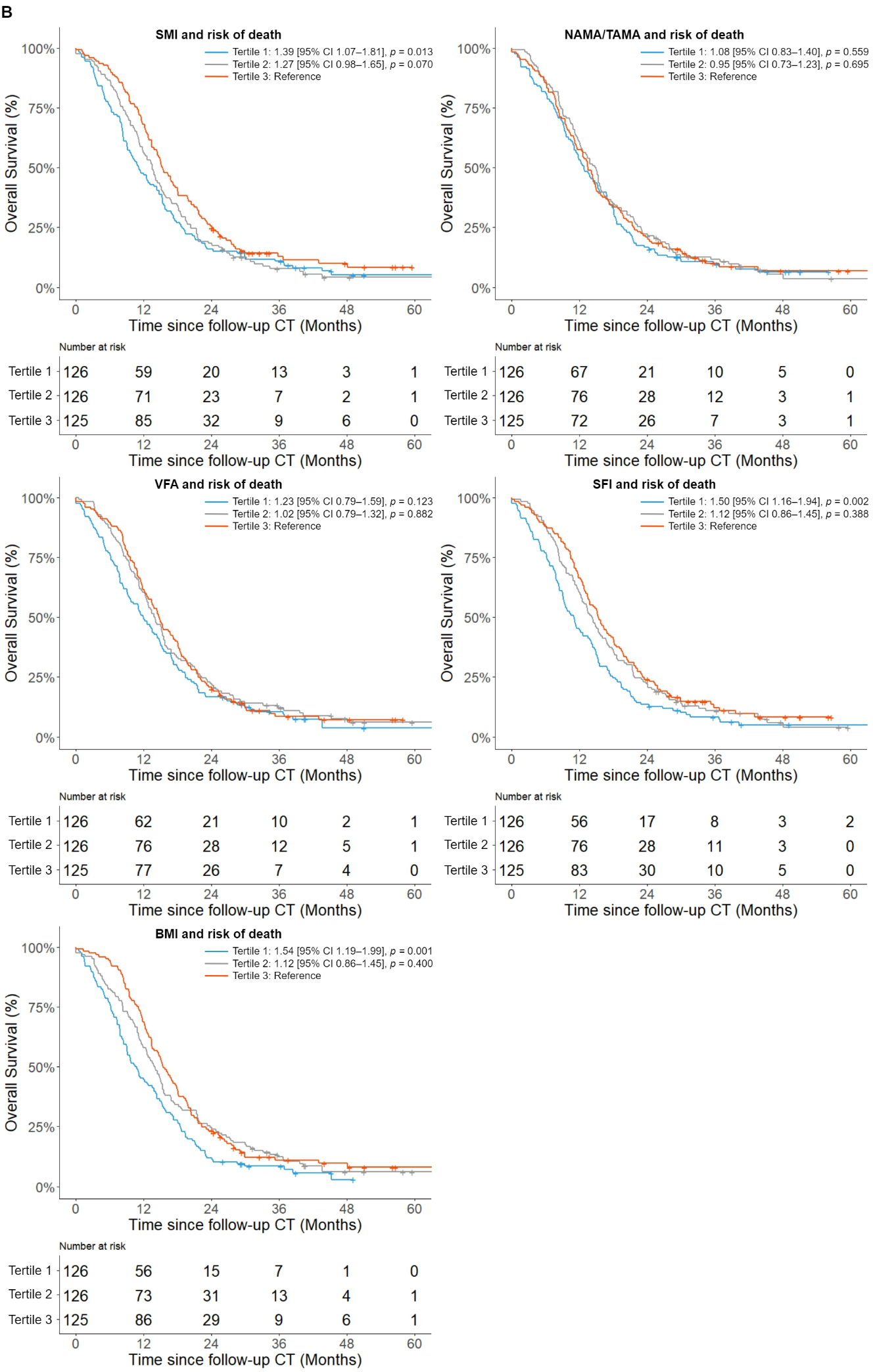
Supplementary Figure 4. Kaplan–Meier curves of OS based on changes in body composition (tertile analysis). (A) curative resection group, (B) no curative resection group.**

Note: Shows survival curves stratified by tertiles of body composition changes from baseline to 12 weeks. Tertile 3 (lesser decrease) is used as the reference group, and separate p-values are provided for comparisons of Tertile 1 (greater decrease) and Tertile 2 (moderate decrease) against Tertile 3.

Abbreviations: SMI, skeletal muscle index; NAMA/TAMA, normal attenuation muscle area/total abdominal muscle area; VFA, visceral fat area; SFI, subcutaneous fat index; BMI, body mass index.
